# Loss of the WNT9a ligand aggravates the rheumatoid arthritis-like symptoms in hTNF transgenic mice

**DOI:** 10.1038/s41419-021-03786-6

**Published:** 2021-05-15

**Authors:** Stefan Teufel, Petra Köckemann, Christine Fabritius, Lena I. Wolff, Jessica Bertrand, Thomas Pap, Christine Hartmann

**Affiliations:** 1 Department of Bone and Skeletal Research, Institute of Musculoskeletal Medicine, Medical Faculty of the Westphalian Wilhelm University, 48149 Münster, Germany; 2 Department of Orthopedic Surgery, Otto-von-Guericke University Magdeburg, 39120 Magdeburg, Germany; 3 Department of Molecular Medicine, Institute of Musculoskeletal Medicine, Medical Faculty of the Westphalian Wilhelm University, 48149 Münster, Germany

**Keywords:** Bone remodelling, Rheumatoid arthritis

## Abstract

Agonists and antagonists of the canonical Wnt signaling pathway are modulators of pathological aspects of rheumatoid arthritis (RA). Their activity is primarily modifying bone loss and bone formation, as shown in animal models of RA. More recently, modulation of Wnt signaling by the antagonist Sclerostin has also been shown to influence soft-tissue-associated inflammatory aspects of the disease pointing towards a role of Wnt signaling in soft-tissue inflammation as well. Yet, nothing is known experimentally about the role of Wnt ligands in RA. Here we provide evidence that altering Wnt signaling at the level of a ligand affects all aspects of the rheumatoid arthritic disease. WNT9a levels are increased in the pannus tissue of RA patients, and stimulation of synovial fibroblasts (SFB) with tumor necrosis factor (TNF) leads to increased transcription of Wnt9a. Loss of *Wnt9a* in a chronic TNF-dependent RA mouse model results in an aggravation of disease progression with enhanced pannus formation and joint destruction. Yet, loss of its activity in the acute K/BxN serum-transfer induced arthritis (STIA) mouse model, which is independent of TNF signaling, has no effect on disease severity or progression. Thus, suggesting a specific role for WNT9a in TNF-triggered RA. In synovial fibroblasts, WNT9a can activate the canonical Wnt/β-catenin pathway, but it can also activate P38- and downregulate NFκB signaling. Based on in vitro data, we propose that loss of Wnt9a creates a slight proinflammatory and procatabolic environment that boosts the TNF-mediated inflammatory response.

## Introduction

Inflammation, together with increased pannus formation, as well as progressive joint and bone destruction, are hallmarks of rheumatoid arthritis (RA), leading to joint malformations. These joints lack any signs of cartilage or bone repair, with the observed bone loss being accredited to dysregulated osteoclastogenesis. Clinically, RA presents a symmetric polyarthritis affecting mostly diarthrodial joints^1^. Due to infiltration of inflammatory cells and enhanced local cellular proliferation, the synovium expands, forming the pannus. The inflamed tissue covers and invades the articular cartilage and adjacent bone and produces proinflammatory cytokines, such as TNF and IL-1β. TNF stimulates, among others, the differentiation of osteoclasts that are responsible for the focal bone erosions in different RA mouse models^2–4^. In mice, transgenic expression of a stabilized mRNA encoding human TNF (hTNFtg) leads to joint alterations, essentially mimicking those found in human RA patients^5^. IL-1β is the key-cytokine in the serum-transfer-induced K/BxN RA (STIA) mouse model^6–9^.

Among others, Wnt signaling is modulated in the joint tissue of human RA patients and hTNFtg mice and has been implicated to play a role in RA^10–16^. Wnt signaling is well known for its roles in bone and cartilage formation as well as in bone homeostasis^17–19^. The WNT protein family encompasses 19 distinct ligands that regulate morphogenesis processes during embryogenesis, stem cell maintenance, tissue homeostasis, and proliferation during embryogenesis and in cancer^20–26^. WNTs can interact with different receptor complexes and signal through diverse pathways, among which the canonical Wnt/β-catenin pathway is best understood^27,28^. Canonical Wnt signaling is characterized by stabilization and nuclear translocation of β-catenin and can be visualized by increased expression of target genes, such as *Axin2*^29^. Noncanonical Wnt signaling results in the activation of very diverse signaling pathways^27^. Wnt signaling is modulated and fine-tuned extracellularly through secreted agonists and antagonists. One of these antagonists, Dickkopf 1 (DKK1), is upregulated in the serum of RA patients and the serum and synovial tissue of hTNFtg mice and has been proposed to be a master regulator in joint remodeling blocking bone formation and promoting bone destruction^11^. Yet, its inhibition in hTNFtg mice did not affect pannus-associated inflammatory aspects of RA^11^. Another antagonist, Wnt inhibitory factor 1 (WIF1), expressed in chondrocytes, is downregulated in experimental arthritis and articular cartilage of RA patients^30–32^. Its loss partially protected hTNFtg mice against bone but not cartilage erosion, the latter being even aggravated, but soft-tissue inflammatory disease aspects were unaffected^32^. Both antagonists are Wnt/β-catenin pathway targets^33^. Treatment of hTNFtg mice with the agonist, R-Spondin 1, protected against bone and cartilage loss, but again soft-tissue inflammation was unaffected^34^. On the other hand, loss of yet another antagonist, Sclerostin (SOST), which is also upregulated in RA biopsies and synovial tissue in the hTNFtg RA mouse model, led to worsening of all disease aspects, including soft-tissue inflammation^35^.

Wnt9a is expressed in the developing synovial joints, and *Wnt9a*-deficient embryos are smaller in size, display temporally delayed chondrocyte maturation and synovial chondromatosis in their elbow joints^36^. Here, we show that WNT9a levels are increased in the pannus of RA patients and that its expression can be induced in mouse SFBs by TNF treatment. Hence, WNT9a may either be involved in disease progression or act as a protective factor. To distinguish between these possibilities, hTNFtg mice lacking *Wnt9a* in the limb mesenchyme were generated, and the STIA model was applied to *Wnt9a*-deficient mice.

## Material and methods

### Human material

Research with human material was performed in accordance with the Declaration of Helsinki. Human synovial tissue was dissected during surgery of RA (*n* = 4) and OA (*n* = 4) patients, fixed in freshly prepared 4% paraformaldehyde/PBS at 4 °C for 24 h, embedded in paraffin, and sectioned at 4 μm. Human synovial fibroblasts were isolated from synovial tissue of RA (*n* = 6) and OA (*n* = 6) patients undergoing joint replacement surgery. RA patients met the American College of Rheumatology criteria. Isolated fibroblasts were cultured using high glucose DMEM supplemented with 10% fetal bovine serum and 1% penicillin/streptomycin at 37 °C, 5% CO_2_ for maximal eight passages before RNA isolation using the Qiagen RNeasy Midi Kit. The Invitrogen SuperScript™ III system was used for first-strand synthesis.

The study was approved by the Institutional Ethical Review Board of the Faculty of Medicine of the Otto-von-Guericke University (IRB No 73/18). Informed consent was obtained from the patients prior to surgery. We included 20 patients undergoing either surgery for TKA implantation due to osteoarthritis or rheumatoid arthritis.

### Animal models

The following mouse strains were used: *R26CreERT2*^37^, *Prx1Cre*^38^, *Wnt9a* floxed and *Wnt9*a germline deleted (*Wnt9a*^+/−^)^36^, *Ctnnb1* (exon 3) floxed^39^, and *hTNFtg* (strain Tg197)^5^ with a mixed 129 Sv/J;C57Bl/6 J background. For the hTNFtg arthritis model, *Wnt9a*^+/−^;*Prx1Cre*;*hTNF*^tg/+^ male mice were bred with *Wnt9a*^fl/fl^ females to generate *hTNF*^tg/+^;*Wnt9a*^fl/−^;*Prx1Cre* (*hTNF*^tg/+^;*Wnt9a*^∆Prx/−^) mice. *hTNF*^tg/+^;*Wnt9a*^fl/+^ or *hTNF*^tg/+^;*Wnt9a*^∆Prx/+^ littermates correspond to *hTNF*^tg/+^ specimens and *Wnt9a*^fl/+^ or *Wnt9a*^∆Prx/+^ littermates to controls. From the age of 5 weeks onward, mice of both genders were scored in a blinded fashion once a week for weight, grip strength, and semi-quantitatively for paw swelling, and finally sacrificed at the age of 6 and 8 weeks, or 12 weeks in the case of *hTNF*^tg/+^ mice. Detailed information of the gender of the mice is provided in the Supplementary data and Supplementary Table S1. For the STIA model, 8-week-old female littermates of the genotypes *Wnt9a*^*∆*Prx1/−^ and *Wnt9a*^∆Prx1/+^ (control) were housed together and injected two times (on days 0 and 2) intraperitoneal with 150 µl of arthritogenic serum collected from K/BxN mice^6^. Mice were scored three times a week for weight, grip strength, and using calibers for ankle width in a blinded fashion, and finally sacrificed 14 or 28 days later. Grip strength was determined in a blinded fashion using a grip strength device (Bioseb) following manufacturers’ instructions. Sample sizes in both models (*n* ≥ 8) were based on previous studies using these models. For cell culture experiments, 7-day-old homozygous *Wnt9a*^fl/fl^, *Wnt9a*^fl/fl^;*R26CreER*, and *Ctnnb1*^fl/fl^;*R26CreER* mice were used. Adult mice were sacrificed by cervical dislocation and juveniles by decapitation. Genotyping was performed using previously published PCR protocols. As animals of specific genotypes were allocated to the different groups, no randomization was performed, and no animals were excluded from the analysis. Animal experiments were performed in accordance with the 10 Essentials of the ARRIVE guideline and relevant regulations under the licenses 84-02.04.2014.A056 and 81-02.04.2017.A409 approved by the national and local authorities.

### Micro-Computed tomography (microCT), histomorphometric analysis, and scoring

Mouse limbs were fixed for 48 h in 4% paraformaldehyde at RT, washed in 70% ethanol, and scanned using the SkyScan 1176 microCT (Bruker) with an 0.5 mm aluminum filter at 50 kV, 500 μA, 8.52 μm image pixel size, and 955 ms exposure. Sections were reconstructed using the NRecon v1.7.4.6 software (SkyScan; Bruker) with beam hardening correction set to 40%. The CT Analyzer v1.18.9.0 software was used for trabecular bone analysis of the 426–2131 μm region below the growth plate. MicroCT images of mouse paws were semi-quantitatively scored for affected areas (0 = none, 1 = few small & localized, 2 = multiple small to medium, 3 = multiple medium to large), degree of erosion (0 = none, 1 = roughness, 2 = pitting, 3 = holes), and bone formation (0 = none, 1 = small osteophytes, 2 = spurs, 3 = bone deformity or fusion), with the total score of 0–9 being the sum of the individual scores. Scanning, reconstruction, and the final scoring was performed in a semi-blinded fashion, as recording was based on the identification-number (ear marking) of the individual animal without direct association to the genotype.

### Beta-galactosidase staining, histology, trap, and immunostainings

For beta-galactosidase staining, hind paws were fixed in 0.2% glutaraldehyde for 18 h at 4 °C and stained for 48 h at 37 °C with X-Gal solution (1 mg/ml X-Gal, 2 mM MgCl, 5 mM potassium ferricyanide, 5 mM potassium ferrocyanide, 0.2% NP-40), followed by decalcification with 10% EDTA/tris-buffered saline before dehydration, embedding in paraffin, sectioning at 5 µm, and counterstaining with nuclear fast red.

For histological analysis and tartrate-resistant-alkaline-phosphatase (TRAP) staining, decalcified and paraffin-embedded hind paws were sectioned at 5 µm. Following deparaffinization and rehydration, sections were stained sequentially for 90 s in 0.02% Fast Green FCF and 30 min in 0.1% Safranin O to assess cartilage defects.

For TRAP, sections were incubated for 20 min with 600 µg/ml Fast Red Violet LB Salt and 100 µg/ml Naphthol ASMX Phosphate in dimethylformamide, buffered with 40 mM Sodium acetate/10 mM Sodium tartrate at pH 5.0, and counterstained with Mayer’s Hematoxylin.

For immunohistochemistry, sections were enzymatically pretreated, incubated in 3% H_2_O_2_ for 30 min, blocked with 10% serum, and incubated with primary antibodies (see Supplementary Table S2). Sections were developed using the appropriate species-specific biotinylated secondary antibody (Vector Laboratories) diluted 1:250 in combination with the Vectastain Elite ABC Kit and Diaminobenzidine, and counterstained with methyl green.

### Isolation and cultivation of primary murine cells

Wild-type deep layer chondrocytes (DLCs) were isolated from the knees (femoral and tibial head) and SFBs from the paws of 7-day-old pups. For this, pups were skinned, and the required parts cleared of soft tissue. To isolate DLCs, knees were predigested with 0.3% trypsin for 1 h. Afterwards, the remaining tissue was treated with 0.5 mg/ml collagenase II for 18 h at 37 °C while shaking. DLCs were cultured in DMEM/F-12 with 10% FCS. For SFB isolation, tarsal and metatarsal elements were dissected and digested with 1 mg/ml collagenase IV (310 u/mg dw) for about 20 min under heavy stirring. Cells were cultured in high glucose DMEM with 10% FCS at 37 °C, 5% CO_2_ up to passage 4/5.

The floxed allele in *Wnt9a*^*fl/fl*^ cells was recombined using a Cre-expressing lentivirus. Controls were infected with a *Cre*-less lentivirus. In *Wnt9a*^*fl/fl*^;*R26CreER*, and *Ctnnb1*^*fl/fl*^;*R26CreER* SFBs, recombination was achieved by 4-hydroxytamoxifen (80 ng/ml) treatment for 48 h. For cytokine treatment, SFBs were exposed either for 6, 8, and 12 h to analyze short-term effects, or 48 h to analyze longer-term effects to the following concentrations: hTNFα (10 ng/ml), mTNFα (10 ng/ml), or mIL-1β (10 ng/ml). For WNT9a gain-of-function experiments, SFBs were stimulated for 5 days or as for the β-catenin-dependent Axin2 assay for 48 h with rWNT9a (300 ng/ml) or rWNT3a (100 ng/ml). To assess the rWNT9a effect on hTNF treatment, cells were stimulated with rWNT9a for 2 h before hTNF application.

### Lentiviral preparation

Calcium-phosphate transfection was performed in HEK-293T cells with the following plasmid mixture: 3.5 µg pMD2-VSVG (Addgene #12259), 6.5 µg psPAx2 (Addgene #12260), and either 10 µg p156RRL-nlsCre (Addgene #12106) or p156RRL-nlsCre^−^ (for control). After 6–8 h, the medium was exchanged with medium of the respective target cell type. Two days later, supernatant was collected, filtrated (0.45 µm), and mixed 1:1 with target cell medium, supplemented with 10 µg/ml polybrene, and applied to target cells. Cells were lysed for RNA isolation 24 h later. Deletion efficiency was detected by qPCR.

### Immunoblots

Whole protein lysates from SFBs were prepared as previously described^40^. 12.5 µg lysate was run on a 10% SDS-PAGE and transferred to a 0.45-μm PVDF-membrane by semi-dry transfer (PerfectBlue™, PeqLab). Membranes were blocked using 5% milk or 4% BSA in TBST, incubated with the appropriate primary (see Supplementary Table S3) followed by the respective species-specific HRP-coupled secondary antibodies, and developed using ECL substrate and hyperfilm film.

### RNA isolation, cDNA synthesis, and qPCR analysis

Total RNA was isolated from cultivated DLCs or SFBs using the RNAqueous Kit. First-strand cDNA synthesis was performed using PrimeScript RT Reagent Kit. For qPCR, cDNA was mixed with PerfeCTa SYBR Green FastMix and respective primers (see Supplementary Table S4) in 25 µl total volume. Gene expression was monitored using a BioRad CFX96 cycler. Reaction conditions were 95 °C 30 s, 45× (95 °C 15 s, 60 °C 30 s, 72 °C 20 s + plate read), 72 °C 5 min, melting curve (55–99 °C), in 0.5 °C increments for 5 s + plate read. Values were calculated using the comparative ∆C(t) method and normalized to two housekeeping genes (*HPRT*, *B2M*, *Hprt*, *Sdha*).

### In vitro osteoclastogenesis assay

For osteoclast cultures, monocytes were isolated by flushing the bone marrow from 8-week-old wild-type mice, incubated for 1 day with minimum essential medium eagle, 10% FCS, and primed towards osteoclastic differentiation using 50 ng/ml M-CSF. The next day, cells in the supernatant were collected, transferred to 96-well plates (2 × 10^5^ cells/well), and cultured for 4 days in the presence of 50 ng/ml M-CSF, 20 ng/ml RANKL, and 20% conditioned medium (CM). CM was collected after three days of culture from *Wnt9a*-deficient or control SFBs and filtered before use. Osteoclasts were detected using a Leukocyte Acid Phosphatase (TRAP) Kit, according to the manufacturer’s instructions.

### Image acquisition

Histological bright-field images were acquired on the Zeiss AxioImager.M2 or Zeiss Observer.Z1 using the AxioCam MRc 6.45 µm color camera and the Zen 2 or Zen 3 Zeiss software (Jena, Germany).

### Statistical analysis

Statistical analysis was performed by two-tailed, unpaired Student’s *t*-test using the GraphPad Prism software 6.0. Data are displayed as mean values ± standard deviation. *P*-values less than 0.05 were considered significant. The minimal number of biological replicates for each experiment was *n* = 3.

Detailed information regarding the vendors and order numbers can be found in Supplementary Table S5.

## Results

### WNT9a levels in synovial fibroblasts are elevated under inflammatory conditions

**Fig. 1:**
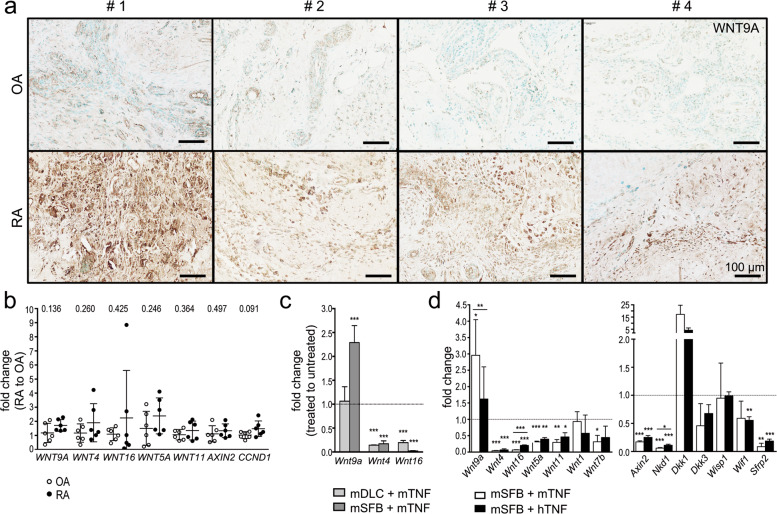
Expression of Wnt genes under inflammatory conditions. **a** Immunohistological analysis of WNT9a in human OA and RA tissue samples (*n* = 4). **b** qPCR analysis of WNT pathway-related genes in RA relative to OA SFB samples displayed as a dot plot with *P*-values noted above (*n* = 6). **c** qPCR analysis of murine *Wnt9a*, *Wnt4*, and *Wnt16* in cultured deep layered chondrocytes (DLC; *n* = 3) and synovial fibroblasts (SFB; *n* = 6) after treatment with murine TNF for 48 h, displayed as relative fold change to untreated cells. **d** qPCR analysis of Wnt and Wnt pathway-related genes in SFBs treated with recombinant murine TNF (mTNF) or human TNF (hTNF) for 48 h, displayed as relative fold change to untreated cells (*n* ≥ 3). *P*-values: *<0.05, **<0.01, and ***<0.001.

Biopsies of human RA patients displayed increased WNT9a levels compared to OA patients (Fig. 1a). Accordingly, *WNT9a* expression was slightly elevated in cultured SFBs of RA relative to OA patients. The expression of other Wnts, which are either expressed in the developing murine synovial joint or upregulated in human RA joints^10,16,31,36,41^, was tendentially increased in cultured RA relative to OA SFBs, as were the *AXIN2* and Cyclin D1 (*CCND1*) levels (Fig. 1b).

Next, we assessed the effects of exogenous TNF on the expression of Wnt pathway components. For this, mouse SFBs and DLCs were treated with either murine (m) or human (h) TNF for 48 h. In SFBs, but not DLCs, Wnt9a expression increased, while Wnt4 and Wnt16 decreased in DLCs and SFBs upon stimulation with mTNF (Fig. 1c,d). In SFBs, the Wnt9a increase in response to hTNF was lower, while all other Wnt genes analyzed either decreased or, as Wnt1, remained nearly unchanged after m/hTNF treatment (Fig. 1d). Yet, in shorter-term treatments, Wnt9a expression was only significantly increased after mTNF treatment for 12 h (Supplementary Fig. 1a), suggesting a secondary mechanism for Wnt9a regulation.

The transcription of Wnt pathway components/regulators was primarily downregulated in mSFBs in response to m/hTNF, with two exceptions, Wisp1 (Wnt1-inducible signaling pathway protein 1) and Dkk1. Wisp1 expression was unaltered, while Dkk1 was elevated (Fig. 1d).

In SFBs, either *Wnt9a*-deficient or treated with recombinant Wnt9a (rWNT9a), Wnt pathway components/regulators were changed as following: Wnt4 and Wnt5a increased slightly in *Wnt9a*-deficient SFBs (Supplementary Fig. 1b). On the other hand, Wnt4, Wnt11, and Sfrp2 decreased, while Axin2 and Wif1 expression increased in response to rWNT9a treatment (Supplementary Fig. 1b). While Axin2 expression was not altered in *Wnt9a*-deficient SFBs, it decreased in *Wnt9a*-deficient DLCs (Supplementary Fig. 1b, c). In certain cells, including chondrocytes, WNT9a can activate the canonical Wnt/β-catenin pathway, but it may also activate noncanonical Wnt pathways^36,42–44^. To analyze whether WNT9a utilizes the Wnt/β-catenin pathway in SFBs, Axin2 levels were examined in *Ctnnb1*-deficient SFBs treated with rWNT9a or rWNT3a (as a control). This resulted in a 10-fold reduction of Axin2 expression in response to either rWNT3a or rWNT9a treatment (Supplementary Fig. 1d), suggesting that Axin2 upregulation in response to rWNT9a is β-catenin-dependent. In RA, according to the literature, opposite effects on canonical Wnt signaling have been reported: β-catenin levels are elevated in the synovium of RA patients, suggesting increased signaling^45^, while the high DKK1 levels in the hTNFtg mouse model imply an overall decrease in signaling. Yet, anti-DKK1 antibody treatment did not affect pannus size^11^. Hence, canonical Wnt signaling in the pannus may be unaffected to a certain extent. Concordantly, BAT-gal activity was increased in the pannus of *hTNF*^tg/+^;BAT-gal mice (Supplementary Fig. 1e), and AXIN2 levels were increased in the *hTNF* ^tg/+^ synovium (Supplementary Fig. 1f). This is somewhat contradictory to the hTNF-induced Axin2 downregulation in SFBs in vitro, yet, cells in the pannus of hTNFtg mice are exposed to TNF signaling for a much longer time.

### Loss of *Wnt9a* aggravates the hTNF-transgene-driven inflammation

**Fig. 2:**
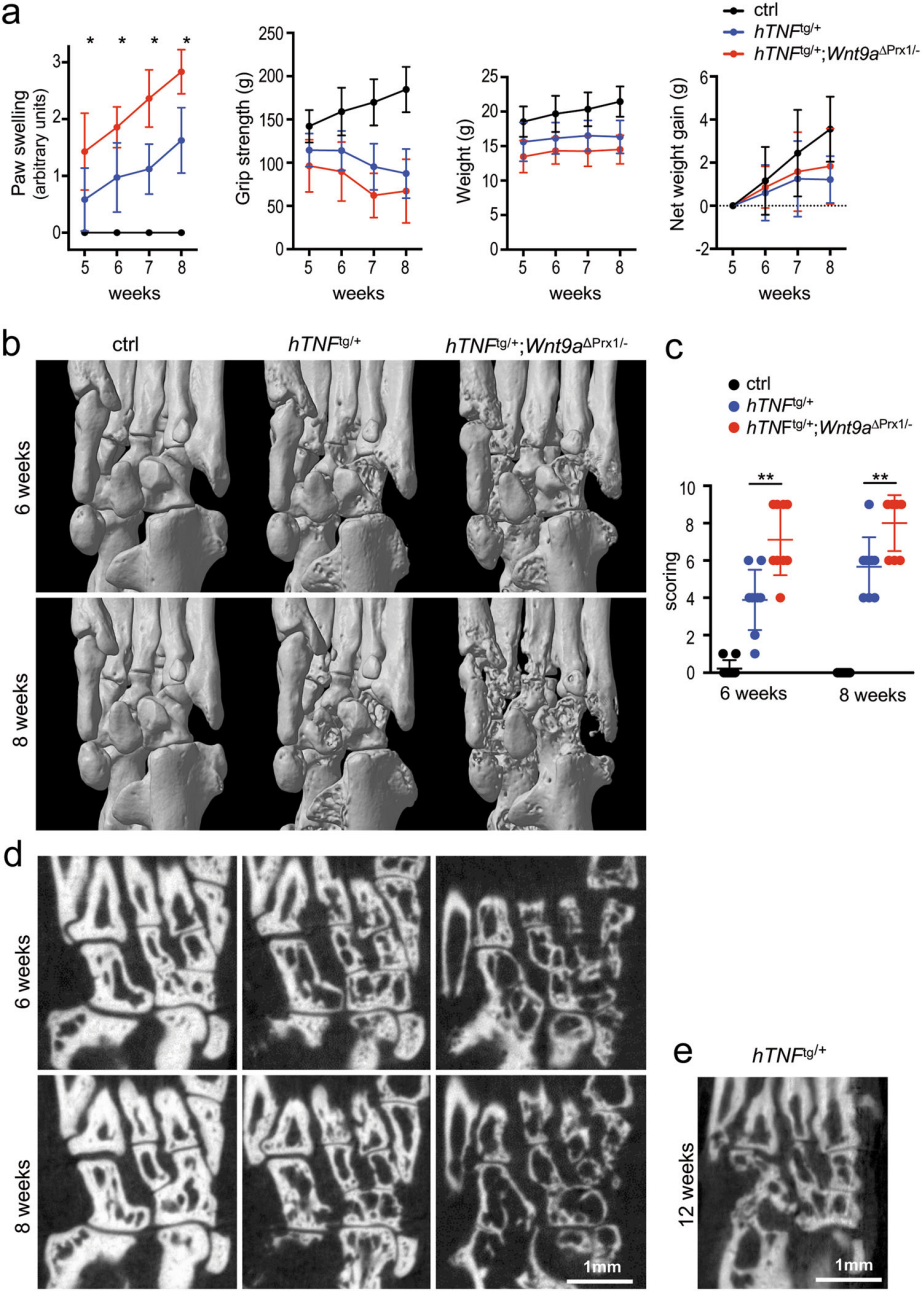
*Wnt9a*-deficiency worsens clinical parameters of experimental arthritis and promotes bone destruction in hTNF transgenic mice. **a** Assessment of disease progression by measurement of paw swelling, grip strength, and weight in control (ctrl), *hTNF*^tg/+^, and *hTNF*^tg/+^;*Wnt9a*^∆Prx/−^ mice over 5–8 weeks. The net weight gain in the *hTNF*^tg/+^and *hTNF*^tg/+^;*Wnt9a*^∆Prx/−^ mice was similar. Data are presented as the mean ± SD (ctrl: *n* = 19; *hTNF*^tg/+^: *n* = 34; *hTNF*^tg/+^;*Wnt9a*^∆Prx/−^: *n* = 21 for the 5- and 6-week timepoints, and ctrl: *n* = 9; *hTNF*^tg/+^: *n* = 25; *hTNF*^tg/+^;*Wnt9a*^∆Prx/−^: *n* = 12, for the 7- and 8-week timepoints). **b** Representative three-dimensional microCT images of ctrl, *hTNF*^tg/+^, and *hTNF*^*t*g/+^;*Wnt9a*^∆Prx/−^ hind paw regions at the 6- and 8-week timepoint (*n* = 9). **c** Scoring of the three-dimensional microCT images with regard to the extent of bone destruction (arbitrary units) (*n* = 9). **d** Two-dimensional representative microCT images of ctrl, *hTNF*^tg/+^, and *hTNF*^tg/+^;*Wnt9a*^∆Prx/−^ hind paw regions at the 6- and 8-week timepoint (*n* = 9). **e** For comparison, a two-dimensional representative microCT image of the *hTN*F^tg/+^ hind paw region at a 12-week timepoint (*n* = 3). *P*-values: *<0.05, **<0.01.

**Fig. 3:**
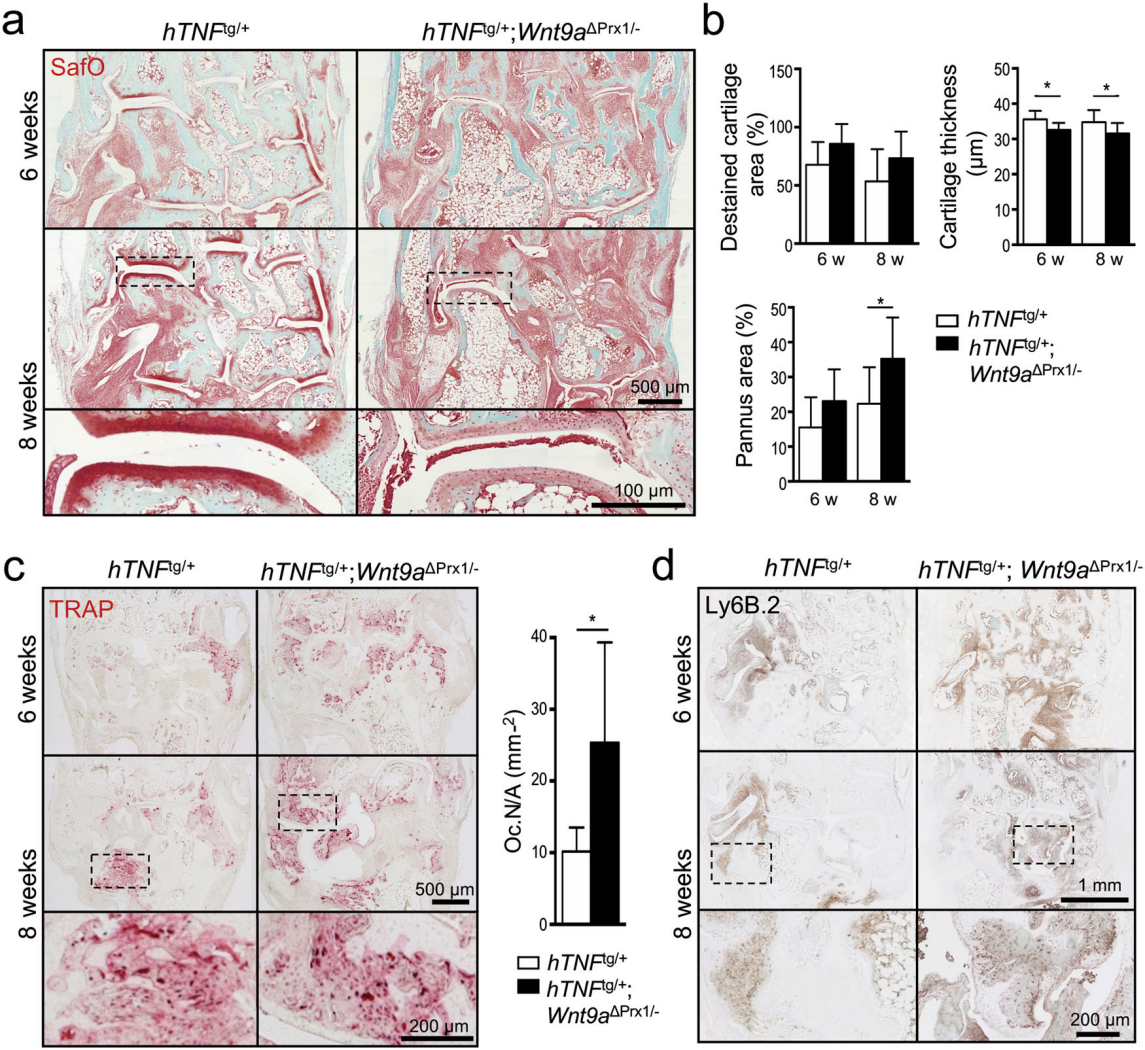
*Wnt9a*-deficiency promotes pannus formation and joint destruction in hTNF transgenic mice. **a** Representative images of Safranin O stained tarsal joint sections from 6- and 8-week-old *hTNF*^tg/+^ and *hTNF*^tg/+^;*Wnt9a*^∆Prx/−^, showing increased pannus formation in the *hTNF*^tg/+^;*Wnt9a*^∆Prx/−^ hind paw regions (*n* = 9). **b** Quantitative histomorphometric analysis of destaining of cartilage, cartilage thickness, and pannus area in 6- and 8-week-old *hTNF*^tg/+^ and *hTNF*^tg/+^;*Wnt9a*^∆Prx/−^ hind paws (*n* = 9). **c** Representative images of tartrate-resistant acid phosphatase (TRAP) staining (red color) of tarsal joint sections from 6- and 8-week-old *hTNF*^tg/+^and *hTNF*^tg/+^;*Wnt9a*^∆Prx/−^ mice (*n* ≥ 4). Quantification of TRAP-positive osteoclasts on sections of 8-week-old *hTNF*^tg/+^ (*n* = 7) and *hTNF*^tg/+^;*Wnt9a*^∆Prx/−^ (*n* = 4) within the tarsal region (as in **a**). **d** Representative images of immunohistochemically stained sections of 6- and 8-week-old *hTNF*^tg/+^ and *hTNF*^tg/+^;*Wnt9a*^∆Prx/−^ hind paws, showing the increased presence of Ly6B.2^+^ neutrophils in the *Wnt9a*-deficient background (*n* = 9). Data in **b** and **c** are presented as mean ± SD, *P*-values: *<0.05.

To address disease progression of the hTNFtg model in a *Wnt9a*-deficient background, we used mice in which the conditional *Wnt9a* allele was deleted in the limb mesenchyme using the *Prx1*-Cre line combined with a germline-deleted *Wnt9a* allele (*hTNF*^tg/+^;*Wnt9a*^∆Prx/−^)^38^. Loss of *Wnt9a* in the limb mesenchyme worsened the clinical signs of inflammation, such as paw swelling and grip strength loss, in the *hTNF*^tg/+^ background (Fig. 2a). Although *hTNF*^tg/+^;*Wnt9a*^∆Prx/−^ mice had a reduced weight compared to *hTNF*^tg/+^ mice, their net weight gain was similar to *hTNF*^tg/+^ mice (Fig. 2a). Radiographic examination revealed significantly increased bone destruction in the hind paws of 6- and 8-week-old *hTNF*^tg/+^;*Wnt9a*^∆Prx/−^ mice compared to *hTNF*^tg/+^ littermates, as quantified in different regions (Fig. 2b–d and Supplementary Fig. 2a, b). Bone destruction in the 8-week-old *hTNF*^tg/+^;*Wnt9a*^∆Prx/−^ mice was even more severe than in 12-week-old *hTNF*^tg/+^ mice (Fig. 2e). Chronic inflammation is associated with a general trabecular bone loss^46^, which also occurred in 8-week-old *hTNF*^tg/+^ mice and was even more pronounced in *hTNF*^tg/+^;*Wnt9a*^∆Prx/−^ mice (Supplementary Fig. 2c). Histopathological examination of the paws revealed an increased inflamed pannus area, being statistically significant at the 8-week timepoint (Fig. 3a, b). Increased cartilage destaining, although not statistically significant, and significant reduction in cartilage thickness were detected in 6- and 8-week-old *hTNF*^tg/+^;*Wnt9a*^∆Prx/−^ mice (Fig. 3b). The paws of *hTNF*^tg/+^;*Wnt9a*^∆Prx/−^ mice also contained more TRAP-positive osteoclasts and neutrophils (Fig. 3c, d). CD45R^+^ B cells were only sparsely detected in some pannus areas of *hTNF*^tg/+^ and *hTNF*^tg/+^;*Wnt9a*^∆Prx/−^ mice (Supplementary Fig. 3a, c). Yet, more and bigger follicular-like B cell clusters were present in the bone marrow of *hTNF*^tg/+^;*Wnt9a*^∆Prx/−^ hind paws (Supplementary Fig. 3a–c).

### Alterations of RA-relevant factors in response to altered Wnt9a signaling

**Fig. 4:**
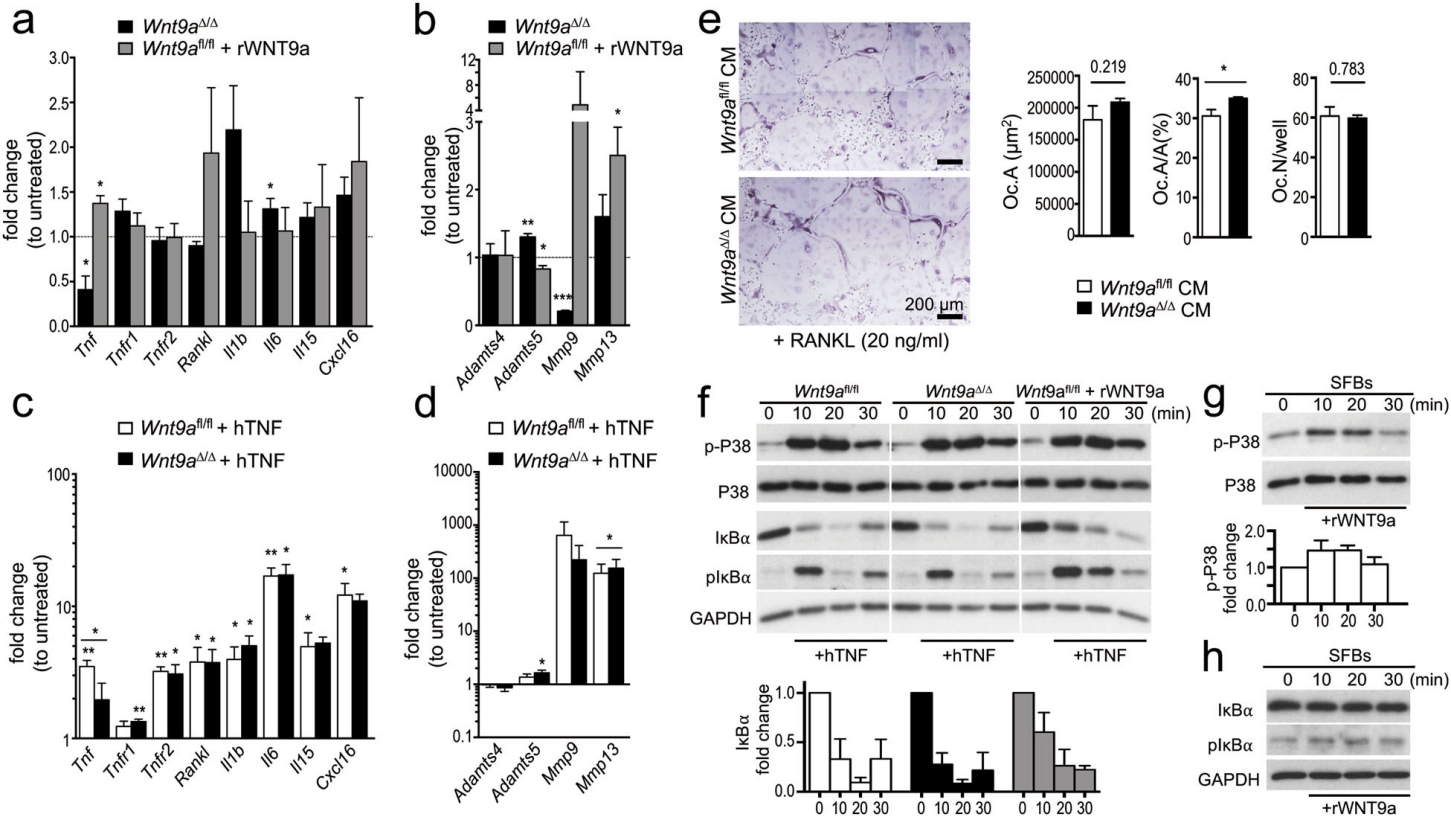
Molecular consequences in synovial fibroblasts after *Wnt9a* deletion or exogenous stimulation with WNT9a or TNF. **a**–**d** qPCR analysis of proinflammatory cytokines, TNF receptors 1 and 2, and Rankl in *Wnt9a*-deficient and wild-type (Wnt9a^fl/fl^) SFBs (**a**, **c**), and of catabolic enzymes involved in cartilage degradation (**b**, **d**) stimulated with rWNT9a for 5 days (**a**, **b**) or treated with hTNF for 48 h (**c**, **d**). Data are displayed as fold expression changes relative to untreated SFBs (*n* = 3). **e** Images of osteoclast differentiation cultures treated for 4 days with conditioned medium (CM) from control (*Wnt9a*^fl/fl^) or *Wnt9a*-deficient SFBs in the presence of 20 ng/ml RANKL; quantified for OC.A (average area covered by a single osteoclast), OC.A/A in % (osteoclast area per total area), and OC.N (osteoclast number per well). **f** Immunoblot for p-P38, P38, IκBα, and GAPDH using extracts from *Wnt9a*^fl/fl^ and *Wnt9a*^∆/∆^ hTNF-treated SFBs, and *Wnt9a*^fl/fl^ SFBs rWNT9a pretreated (2 h) followed by hTNF treatment for the indicated time; below the quantification of IκBα relative to GAPDH levels. **g** Immunoblot for p-P38 and P38 using extracts from rWNT9a-treated SFBs; below the quantification of the p-P38 relative to P38 levels. **h** Immunoblot for IκBα, pIκBα, and GAPDH (loading control) using extracts from rWNT9a-treated SFBs. (**a**–**h**) *n* = 3. *P*-values: *<0.05, **<0.01.

Activated synovial fibroblasts are considered to be the main culprits in RA, and TNF levels are particularly high in these cells in the hTNFtg RA mouse model. Therefore, we addressed whether the expression of RA-relevant factors is altered in *Wnt9a-*deficient SFBs. Regarding proinflammatory cytokines, Tnf expression was decreased, while transcriptional levels of Il1b, Il6, Il15, and Cxcl16 were slightly increased (Fig. 4a). Of the catabolic enzymes, the expression of Adamts4 was unchanged, Adamts5 and Mmp13 slightly increased, while Mmp9 was significantly decreased (Fig. 4b). Interestingly, rWNT9a treatment of wild-type SFBs increased Tnf, Rankl, Il15, and Cxcl16 expression (Fig. 4a). Concerning the catabolic enzymes, rWNT9a treatment decreased Adamts5, while increasing Mmp9 and Mmp13 expression (Fig. 4b). The transcriptional levels of two other cytokines, Il17a and Il9, were very low in SFBs and did not change in response to either deletion of Wnt9a or stimulation with rWNT9a (data not shown). Similar alterations in the expression levels of Il1b, Il6, Adamts5, and Mmp13 were also observed in *Ctnnb1*-deficient SFBs independent of the presence or absence of WNT9a stimulation (Supplementary Fig. S4a). Consequently, loss of WNT9a or β-catenin activity in SFBs fosters a slight proinflammatory and procatabolic environment. Next, we asked if hTNF treatment would lead to differential effects in *Wnt9a*^fl/fl^ versus *Wnt9a*^∆/∆^ SFBs. Except for a decreased Tnf expression and slight increases in Tnfr1, Il1b, Adamts5, and Mmp13 levels, the expression of the analyzed genes did not change (Fig. 4c, d). As more osteoclasts are present in the *hTNF*^tg/+^;*Wnt9a*^∆Prx/−^ hind paws, we asked whether *Wnt9a*-deficient SFBs produce a secreted factor stimulating osteoclast differentiation. Conditioned medium from *Wnt9a*^∆/∆^ SFBs in presence of 20 ng/ml RANKL led to an increase in the area covered by osteoclasts, while the average number of osteoclasts per well did not change (Fig. 4e).

Next, we asked if loss of *Wnt9a* or treatment with rWNT9a would alter TNF activated signaling pathways, such as P38 and NFκB^47^. Loss of *Wnt9a* did not affect P38 activation upon TNF stimulation (Fig. 4f). Yet interestingly, phospho-P38 (p-P38) levels increased in response to rWNT9a application (Fig. 4g). Nevertheless, rWNT9a pretreatment of SFBs for 2 h before hTNF application did not alter p-P38 levels (Fig. 4f). TNF-induced degradation of the NFκB inhibitor IκBα was also not affected by the loss of *Wnt9a* (Fig. 4f). Yet, pretreatment with rWNT9a delayed IκBα degradation (Fig. 4f), while rWNT9a treatment by itself did not affect IκBα or pIκBα levels in SFBs (Fig. 4h). Thus, WNT9a can, like WNT4, interfere with NFκB signaling and like other WNTs it can activate p38^48–50^.

### Loss of *Wnt9a* does not alter disease severity in the acute serum-transfer RA model

**Fig. 5:**
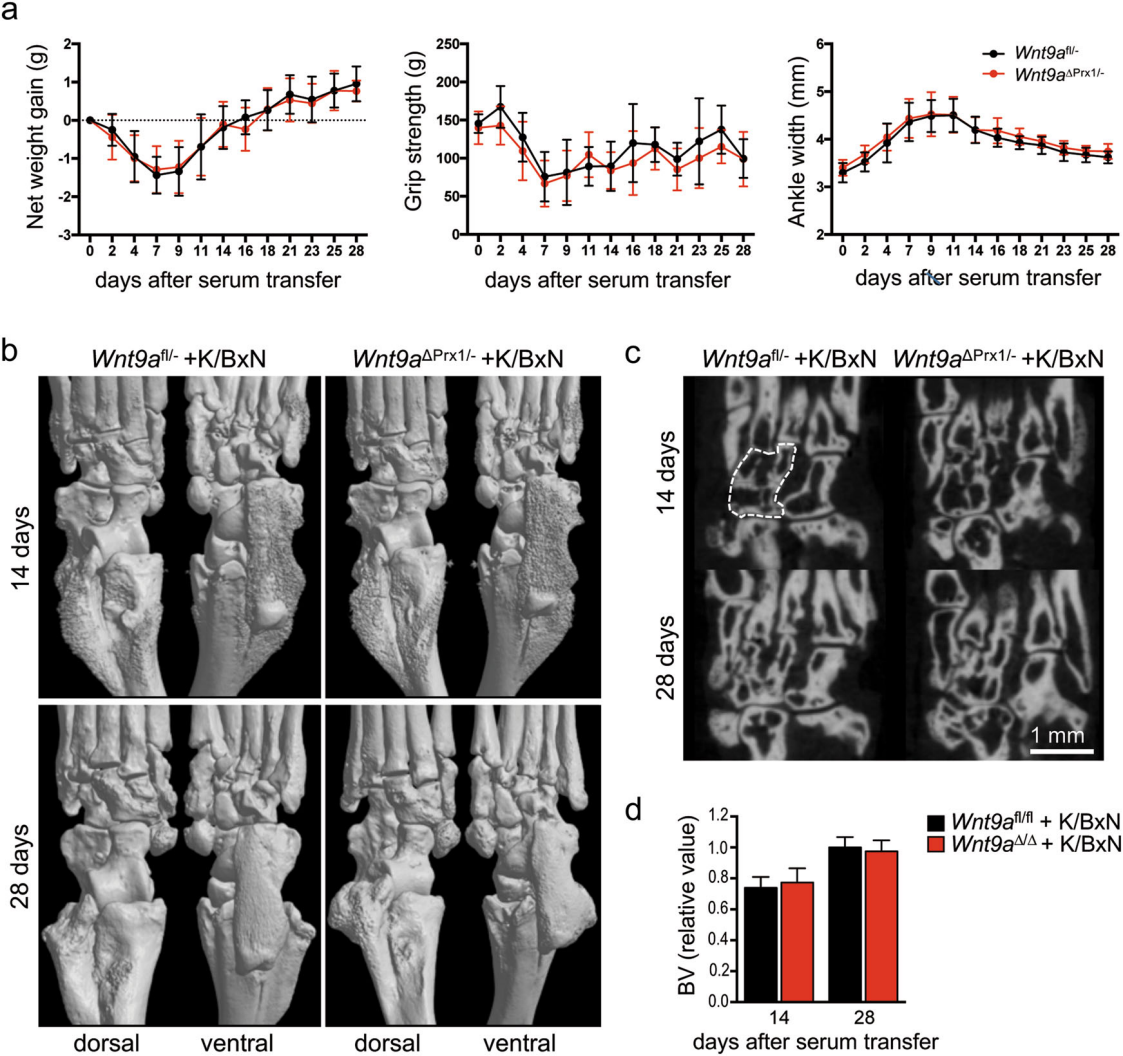
*Wnt9a*-deficiency has no effect on disease parameters or bone and joint destruction in the acute K/BxN STIA model. **a** Assessment of net weight gain, grip strength, and ankle width in K/BxN-treated control (*Wnt9a*^fl/−^) and *Wnt9a*-deficient mice over 28 days. **b** Three- and **c** two-dimensional microCT images of *Wnt9a*^fl/−^ (control) and *Wnt9a*-deficient hind paws, 14 (*n* = 19) and 28 days (*n* = 9) after K/BxN serum transfer. **d** Quantification of the bone volume in the area outlined by the white dashed line in **c**, encompassing the navicular, intermediate, and lateral cuneiform bones, at the 14- (*n* = 10) and 28-day (*n* = 9) timepoint.

**Fig. 6:**
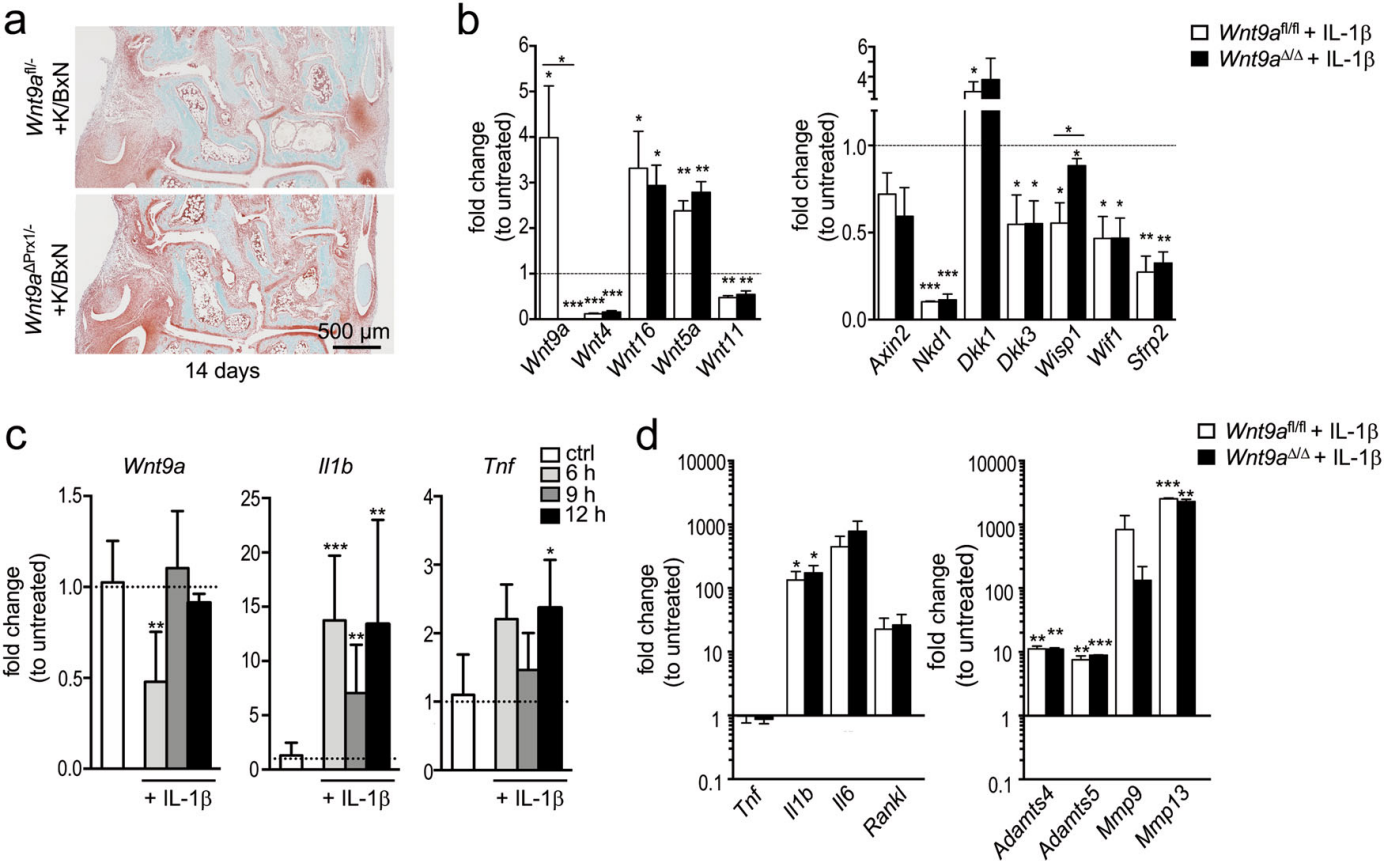
Histological assessment of K/BxN-treated mice and alterations in gene expression levels in *Wnt9a*^fl/fl^ and *Wnt9a*-deficient cells in response to IL-1β treatment. **a** Representative images of Safranin O stained tarsal joint sections of the 14-day timepoint (*n* = 3). **b** qPCR analysis of Wnt genes and Wnt pathway-related genes in *Wnt9a*^*f*l/fl^ and *Wnt9a*-deficient SFBs after treatment with recombinant murine IL-1β. **c** qPCR analysis of short-term effects on the expression of Wnt9a, Il1b, and Tnf in response to stimulation with recombinant IL-1β. **d** qPCR analysis of long-term effects on the expression of cytokines and catabolic enzymes in *Wnt9a*^fl/fl^ (wild-type) and *Wnt9a*-deficient SFBs in response to stimulation with recombinant IL-1β for 48 h.

Next, we asked whether the loss of *Wnt9a* affects the phenotype of the STIA model, driven primarily by autoantibodies and IL-1β as the main cytokine. Here, two timepoints were analyzed, 14 days (peak of the arthritic-like phenotype) and 28 days (regression of the arthritic-like phenotype) after serum transfer^35^. Based on clinical parameters, loss of *Wnt9a* in the mesenchymal limb cells did neither affect the severity of bone destruction in the STIA mouse model, nor disease regression. Net weight gain, grip strength, and paw swelling were very similar between the arthritic *Wnt9a*^fl/−^ control and *Wnt9a*^∆Prx1/−^ mice (Fig. 5a). Concurrently, the extent of bone destruction and reversal thereof was also very similar according to the radiographic images of hind paws from arthritic *Wnt9a*^fl/−^ control and *Wnt9a*^∆Prx1/−^ mice at the 14- and 28-day timepoint (Fig. 5b). This was confirmed by the analysis and quantification of the bone volume (based on µCT-sections of hind paws at the two timepoints) (Fig. 5c,d). Also histologically, no differences were detected between the K/BxN-treated *Wnt9a*^fl/−^ control and K/BxN-treated *Wnt9a*^∆Prx1/−^ mice at the 14-day timepoint (Fig. 6a). Next, we asked whether Wnt9a expression in SFBs was altered by a 48 h IL-1β treatment. By qPCR, Wnt9a was upregulated in SFBs upon treatment with 10 ng IL-1β (Fig. 6b). Yet, short-term IL-1β treatment led to a transient downregulation of Wnt9a at the 6 h timepoint (Fig. 6c). Of the other Wnt pathway-related genes, Wnt16 and Wnt5a were both upregulated in response to IL-1β treatment (Fig. 6b). All other Wnt pathway-related genes behaved similarly to TNF treatment except for Wisp1, which was downregulated upon IL-1β treatment (Fig. 6b). Except for Axin2, these expression changes were all statistically significant. Interestingly, in *Wnt9a*-deficient SFBs only Wisp1 expression was altered after IL-1β treatment; here, the downregulation in response to IL-1β was diminished (Fig. 6b). Consistent with reports on autoregulation and regulation of Il6 and Rankl, IL-1β treatment of SFBs upregulated its own expression as well as the expression of Il6 and Rankl (Fig. 6c, d)^51–53^. Furthermore, IL-1β upregulated Tnf expression at the early timepoints, but not after 48 h of exogenous treatment (Fig. 6c, d). Consistent with the literature, IL-1β treatment increased the expression of catabolic proteolytic enzymes, such as Mmp9, Mmp13, Adamts4, and Adamts5 in wild-type SFBs (Fig. 6d)^54,55^. In the *Wnt9a*-deficient SFBs, the expression of these genes was not different, except for Mmp9, which was slightly, but not statistically significantly downregulated (Fig. 6d).

## Discussion

In contrast to previous investigations regarding the role of Wnt signaling in the experimental hTNFtg mouse model, such as the inhibition of the antagonist DKK1, the loss-of the antagonist WIF1, or treatment with the agonist R-Spondin 1, where soft-tissue inflammatory aspects remained unaffected^11,32,34^, all aspects of the RA-like symptoms were affected by the loss of *Wnt9a*. Furthermore, disease progression and severity were augmented in the *hTNF*^tg/+^;*Wnt9a*^∆Prx/−^ mice. This phenotype is similar to the alterations observed following the loss of the Wnt inhibitor sclerostin^35^. Yet, sclerostin levels remained unaltered in *Wnt9a*-deficient SFBs with or without TNF, or SFBs treated with rWNT9a, and in *hTNF*^tg/+^;*Wnt9a*^∆Prx/−^ mice (Supplementary Fig. 1g, and data not shown).

**Fig. 7:**
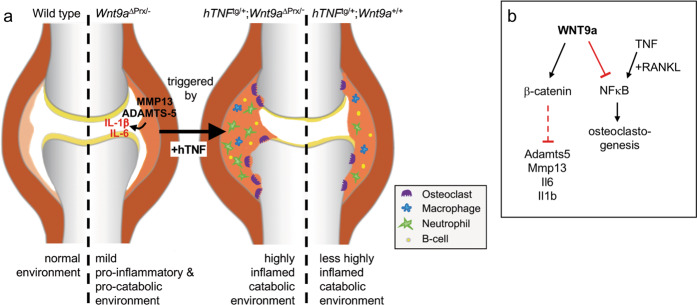
Model comparing the alterations in joints of wild-type versus *Wnt9a*-deficient, *hTNF*^tg/+^;*Wnt9a*^∆Prx/−^, and *hTNF*^tg/+^ animals and pathways utilized by WNT9a to control environmental changes in the joint. **a** Compared to the situation in the wild-type, *Wnt9a*-deficient joints display a mild proinflammatory (slightly increased transcriptional levels of Il1b and Il6) and procatabolic (slightly increased transcriptional levels of Mmp13 and Adamts5) environment. In the *hTNF*^tg/+^;*Wnt9a*^∆Prx/−^ joints, this is converted into a highly inflamed catabolic environment through the activity of TNF. Due to the predispositioning in the *Wnt9a*-deficient joints, the effects of the TNF transgene are more intense compared to the alteration in *hTNF*^tg/+^ joints. **b** WNT9a acts on the one hand through the Wnt/β-catenin pathway to maintain low transcriptional levels of the proinflammatory factors Il1b and Il6, and of the procatabolic factors Adamts5 and Mmp13 in synovial fibroblasts. On the other hand, WNT9a dampens the activity of the transcription factor NFκB, which is required for osteoclastogenesis downstream of TNF and RANKL.

As shown in previous studies, the mild TNF-induced inflammatory response can be dramatically boosted by co-administration of IL-1β^56^. Thus, the modestly increased transcriptional levels of the cytokines IL-1β and IL-6, as observed in *Wnt9a*- and *Ctnnb1-*deficient SFBs, may provide a sensitized proinflammatory environment that could markedly enhance the inflammatory effect of the hTNF transgene and thereby accelerate disease progression (Fig. 7a). Joints of 3-month-old *Wnt9a*^∆Prx1/−^ mice do not show any inflammatory alterations (data not shown), which could partly be due to the observed downregulation of endogenous Tnf expression in the *Wnt9a*-deficient SFBs. We also noted a slight upregulation of the expression of the catabolic enzymes ADAMTS5 and MMP13 in the *Wnt9a*- as well as *Ctnnb1*-deficient SFBs. Both enzymes degrade cartilage matrix and, based on genetic experiments, are essential for cartilage degradation in murine osteoarthritis and rheumatoid arthritis models^54,57,58^. Hence, the in vitro observations are pointing to the presence of a procatabolic environment in the *Wnt9a*^∆Prx1/−^ mice. In combination with the proinflammatory environment, this may predisposition the articular cartilage even further for destruction in the presence of the hTNF transgene. In support of this notion, in 6-month old *Wnt9a*^∆Prx/−^ specimens ADAMTS5- and MMP13-positive cells were observed in the synovium (Supplementary Fig. 4b, c), and 9-month and older *Wnt9a*^∆Prx/−^ mice develop osteoarthritis-like changes in their joints (C.H. and S.T. manuscript in preparation). Our in vitro results suggest that this deregulation of the procatabolic/proinflammatory environment is dependent on β-catenin (Fig. 7b).

Interestingly, these potential predispositioning parameters also exist in the *Wnt9a*^∆Prx1/−^ STIA RA model. Yet surprisingly, the loss of *Wnt9a* did not influence disease progression or severity in this model. In contrast to the hTNFtg model, STIA is a transient RA model being driven by autoantibodies against the glucose-6-phosphate isomerase and mimicking primarily the effector phase of RA^59^. IL-1β signaling is crucial in this model, while TNF plays only a partial, not yet fully understood role^7^. In vitro in SFBs, we observed no upregulation of Tnf expression in response to long-term IL-1β treatment, and in *Wnt9a*-deficient SFBs, the endogenous Tnf expression levels were even reduced. Hence, one could speculate that this may in part be responsible for the observation that the absence of WNT9a-activity in the STIA model does not lead to an aggravation of the phenotype, as the TNF levels may be too low. Alternatively, the time the *Wnt9a*-deficient cells are exposed to inflammatory cytokines may be too short in the acute STIA model, which only exhibits a temporary RA-phenotype. The latter, could be addressed in the future by establishing the chronic STIA model in *Wnt9a*^∆Prx/−^ mice, similarly to what has been observed regarding ApoE-deficiency in combination with the STIA model^60^. Furthermore, in vitro, we detected differences between IL-1β- and TNF-treated SFBs concerning other Wnts, and this may also contribute to the observed differences between the two models.

Based on the results from the *hTNF*^tg/+^;*Wnt9a*^∆Prx^ mice our data suggest that the observed WNT9a upregulation in the synovium of human RA biopsies occurs likely as a protective response. As shown here, exogenous WNT9a can interfere with NFκB signaling in vitro. Hence, one possible mechanism how the in vivo observed upregulation of WNT9a may act in a protective fashion is by negatively influencing TNF-NFκB-driven osteoclastogenesis (Fig. 7b). Along this line, we noted that the number of osteoclasts was increased in the *hTNF*^tg/+^;*Wnt9a*^∆Prx^ mice, and that conditioned medium from *Wnt9a*-deficient SFBs altered the spreading of osteoclasts in vitro in an M-CSF/RANKL mediated osteoclastogenesis. Interestingly, TNF can, in contrast to IL-1β, stimulate osteoclastogenesis even in the absence of Rankl^61^.

Nevertheless there are still some limitations to this study: (a) the expression changes were only analyzed in *Wnt9a* in-vitro-deleted SFBs, which were exogenously treated with TNF, instead of primary cells from the animals, which have a different history, and (b) WNT9a is produced in vivo by other cell-types besides SFBs, such as chondrocytes and osteoblasts, which are also targeted by the *Prx1*-Cre line and may contribute to the phenotype.

Supplementary information is available at Cell Death & Disease’s website.

## Supplementary information

Supporting Information

## Data Availability

Representative data of datasets generated or analyzed during this study are included in this published article and its supplementary information files. All individual datasets used and/or analyzed during the current study are available from the corresponding author on reasonable request.
