# Supplementary material for: Loss of the WNT9a ligand aggravates the rheumatoid arthritis-like symptoms in hTNF transgenic mice
Source: Cell Death Dis. 2021 May 15;12(5):494. doi: 10.1038/s41419-021-03786-6 (PMC8121832; doi:10.1038/s41419-021-03786-6)
Supplement: Supplementary file 1 — Supporting Information [file 41419_2021_3786_MOESM1_ESM.pdf]

Supporting Information

Loss of the WNT9a ligand aggravates the rheumatoid arthritis-like symptoms in hTNF transgenic animals

Stefan Teufel[1], Petra Köckemann[1], Christine Fabritius[1], Lena I. Wolff[1], Jessica Bertrand[2], Thomas Pap[3], Christine Hartmann[1,*]

For the phenotypic scoring, performed in a blinded fashion, described in Figure 2a the gender-composition of the different groups was as following: control-group (13 males; 6 females); TNF^{tg+} group (19 males; 15 females); $TNF^{tg+;}Wnt9a^{\Delta Prx/-}$ group (9 males; 12 females). Whenever possible, littermates of the same sex were housed together.

Supplementary Table S1 Gender of animals included in the microCT and histological analysis regarding the TNF-dependent RA-mouse model (data shown in Fig. 2b-d, Suppl. Fig. S2, Fig. 3, Supp. Fig. S3)

	6 weeks		8 weeks	
	male	female	male	female
ctrl	6	3	7	2
TNF^{tg+}	4	5	5	4
$TNF^{tg+;}Wnt9a^{\Delta Prx/-}$	4	5	4	5

Supplementary Table S2 Antibodies, Dilution and Antigen Retrieval for Immunohistochemistry

Antibody	Order number, Supplier	Dilution	Antigen retrieval
Anti-ADAMTS5	ab231595, Abcam	1:200	Trypsin, 10 min, 37°C
Anti-AXIN2	PA5-25331, Invitrogen	1:100	Trypsin, 10 min, 37°C
Anti-CD45R	550286, BD Biosciences	1:200	20 µg/ml Proteinase K, 10 min, 37°C
Anti-MMP13	ab219620, Abcam	1:20	Trypsin, 10 min, 37°C
Anti-LY6B.2	MCA771GT, BIO-RAD	1:10.000	20 µg/ml Proteinase K, 10 min, 37°C
Anti-SCLEROSTIN	AF1589, R&D	1:100	0.7% HCl, 7min, 37°C, 0.02 mg/ml Pepsin (Sigma) in 0.7% HCl, 45 min
Anti-WNT9a	MAB31571, R&D	1:20	Tris-EDTA, 2x 10 min, ca. 90°C

Supplementary Table S3 Antibodies, Dilutions, and Blocking Conditions for Western Blot

Antibody	Order number, Supplier	Dilution	Blocking
Anti-p38	9212, Cell Signaling	1:1.000	4% BSA
Anti-phosphop38	9211, Cell Signaling	1:1.000	4% BSA
Anti-IκBα	4814, Cell Signaling	1:1.000	4% BSA
Anti-phospho-IκBα	2859, Cell Signaling	1:1.000	4% BSA
Anti-GAPDH	MA5-15738, Thermo Fisher	1:10.000	5% Milk

Supplementary Table S4 Information regarding primer sequences and product length for real-time PCR.

Gene	Sequence	Product size (bp)	Exon-Intron Spanning
AXIN2	5'-GAGTGGACTTGTGCCGACTTCA-3'<br>5'-GGTGGCTGGTGCAAAGACATAG-3'	189	Yes
CCND1	5'-AGTTGTTGGGGCTCCTCAG-3'<br>5'-AGACCTTCGTTGCCCTCTGT-3'	120	Yes
WNT4	5'-CCTTCGTGTACGCCATCTCT-3'<br>5'-GCCTCATTGTTGTGGAGGTT-3'	250	Yes
WNT5a	5'-CGCCCAGGTTGTAATTGAAG-3'<br>5'-GCATGTGGTCCTGATACAAGT-3'	164	Yes
WNT9a	5'-TCGAGTGCCAGTTCCAGTTC-3'<br>5'-AAGGCAGTCTCCTTGAAGCC-3'	100	Yes
WNT11	5'-GTGAAGGACTCGGAACTCGT-3'<br>5'-CTTCCGTTGGATGTCTTGTTG-3'	116	Yes
WNT16	5'-TTCCCCATCGGAAACACCAC-3'<br>5'-GACATCAACTTGGCGACAGC-3'	101	Yes
GAPDH	5'-AATGACCAGTCAACAGGGGAC-3'<br>5'-CCTGACCAAGGAAAGCAAAGTC-3'	135	Yes
B2M	5'-AGATGAGTATGCCTGCCGTG-3'<br>5'-TCATCCAATCCAAATGCGGC-3'	120	Yes
Adamts4	5'-ACAACCACCGAACCGACCTC-3'<br>5'-ATGATCCGGTCACAGCCAGC-3'	229	Yes
Adamts5	5'-TGGTCCAAATGCACGTCAGC-3'<br>5'-GCACAGACATCCATGCCAGG-3'	191	Yes
Axin2	5'-AAGCCTGGCTCCAGAAGATCACAA-3'<br>5'-TTTGAGCCTTCAGCATCCTCCTGT-3'	134	Yes
Cxcl16	5'-GAAGCCAAGACCAGTGGGT-3'<br>5'-GGTACTGGCTTGAGGCAAATG-3'	113	Yes
Dkk1	5'-GGAAATTGAGGAAAGCATC-3'<br>5'-CAGATCTTGGACCAGAAGTG-3'	192	Yes

Dkk3	5’-CGAGAGGTGGAGGAGCTGATG-3’<br>5’-GTCTCCGTGCTGGTCTCATTG-3’	152	Yes
Il1b	5’-CCACCTTTTGACAGTGATGAG-3’<br>5’-CATCAGGACAGCCCAGGTC-3’	101	Yes
Il6	5’-CCACTTCACAAGTCGGAGGC-3’<br>5’-GCAAGTGCATCATCGTTGTTC-3’	112	Yes
Il9	5’-GTCTCTCCGTCCCAACTGATG-3’<br>5’-TCTGTGTGGCATTGGTCAGC-3’	78	Yes
Il15	5’-CATCCATCTCGTGCTACTTGTG-3’<br>5’-CATCTATCCAGTTGGCCTCTG-3’	126	Yes
Il17a	5’-AACACTGAGGCCAAGGACTT-3’<br>5’-ACCCACCAGCATCTTCTCG-3’	324	Yes
Mmp9	5’-ACGACATAGACGGCATCCAGTATC-3’<br>5’-AGGTATAGTGGGACACATAGTGGG-3’	122	Yes
Mmp13	5’-TTCTGGTCTTCTGGCACACGCTTT-3’<br>5’-CCAAGCTCATGGGCAGCAACAATA-3’	132	Yes
Nkd1	5’-CTACCTAGACCTGGCGGGGA-3’<br>5’-GAGAGCGAGTGGGGTTGGAG-3’	113	Yes
Rankl	5’-GGCCACAGCGCTTCTCAG-3’<br>5’-GAGTGACTTTATGGGAACCCGAT-3’	143	Yes
Sfrp2	5’-CCAAGAATGAGGACGACAACG-3’<br>5’-CGCCGTTCAGCTTGTAAATGG-3’	150	Yes
Tnf	5’-TCTCATCAGTTCTATGGCCC-3’<br>5’-GGGAGTAGACAAGGTACAAC-3’	212	Yes
Tnfr1	5’-ATCCCGTGCCTGTCAAAGAG-3’<br>5’-TAAAGCCTGGGGTGCTGAAG-3’	117	Yes
Tnfr2	5’-TACTGCGCCTTGAAAACCCA-3’<br>5’-CCAGGATGCTACAGATGCGG-3’	196	Yes
Wif1	5’-GCTCTGGAGCATCCTACCTT-3’<br>5’-AATCATGTGTAAAGGGGGCC-3’	170	Yes
Wisp1	5’-GTCCTGAGGGTGGGCAACAT-3’<br>5’-GGGCGTGTAGTCGTTTCCTCT-3’	98	Yes

Wnt4	5'-GGCCATCGAGGAGTGCCAAT-3'<br>5'-CGTGCACTGTCCGGTCACA-3'	212	Yes
Wnt5a	5'-GAAGCAGGCCGTAGGAC-3'<br>5'-AGCCAGCACGTCTTGAGG-3'	92	No
Wnt7b	5'-GCGTCCTCTACGTGAAGCTC-3'<br>5'-TCTTGTTGCAGATGATGTTGG-3'	72	Yes
Wnt9a	5'-TGCCAGTACCAGTTCCGCTTT-3'<br>5'-GATGGCGTAGAGGAAAGCAGT-3'	108	Yes
Wnt10b	5'-AATGCGGATCCACAACAAC-3'<br>5'-CTCCAACAGGTCTTGAATTGG-3'	111	Yes
Wnt11	5'-CAGGATCCCAAGCCAATAAA-3'<br>5'-TCCAGGGAGGCACGTAGA-3'	76	Yes
Wnt16	5'-GCCACTACCACTTCCACCC-3'<br>5'-GAGCCACCATTCTGCAAGG-3'	188	Yes
Hprt	5'-AGCTACTGTAATGATCAGTCAACG-3'<br>5'-AGAGGTCCTTTTCACCAGCA-3'	200	Yes
Sdha	5'-GCTCCTGCCTCTGTGGTTGA-3'<br>5'-AGCAACACCGATGAGCCTG-3'	136	Yes

Supplementary Table S5

Detailed order information concerning reagents in alphabetical order

**Reagent**	**Company**	**Order number**
Collagenase II	Worthington	LS004176
Collagenase IV	Worthington	LS004188
Diaminobenzidine	Sigma	D5905
DMEM/F-12	Sigma-Aldrich	D8437
ECL substrate	Amersham	RPN2106
Fast Green FCF	J.T.Baker	M377-03

Fast Red Violet LB Salt	Sigma	F3381
High Glucose DMEM (human SFBs)	Sigma-Aldrich	D0819
High glucose DMEM (murine SFBs)	Sigma-Aldrich	D6429
4-hydroxytamoxifen	Sigma	H6278
Hyperfilm film	Amersham	28906837
RNeasy Midi Kit	Qiagen	75144
Invitrogen SuperScript™ III system	ThermoFisher Scientific	18080051
mIL-1β	R&D	401-ML
M-CSF	R&D	416-ML
Minimum essential medium eagle	Sigma	M4526
Naphthol ASMX Phosphate	Sigma	N5000
PrimeScript RT Reagent Kit	TaKaRa	RR037
PerfeCTa SYBR Green FastMix	Quanta	95072
PVDF-membrane	ThermoFisher Scientific	88518
RANKL	R&D	462-TEC-010
RNAqueous Kit	Invitrogen	AM1912
Safranin O	Sigma	S-8884
TRAP Kit	Sigma	387A
hTNFα	R&D	210-TA-005
mTNFα	R&D	410-MT-010
Vectastain Elite ABC Kit	Vector Laboratories	PK-6100
rWNT3a	R&D	1324-WN
rWNT9a	R&D	8148-WN

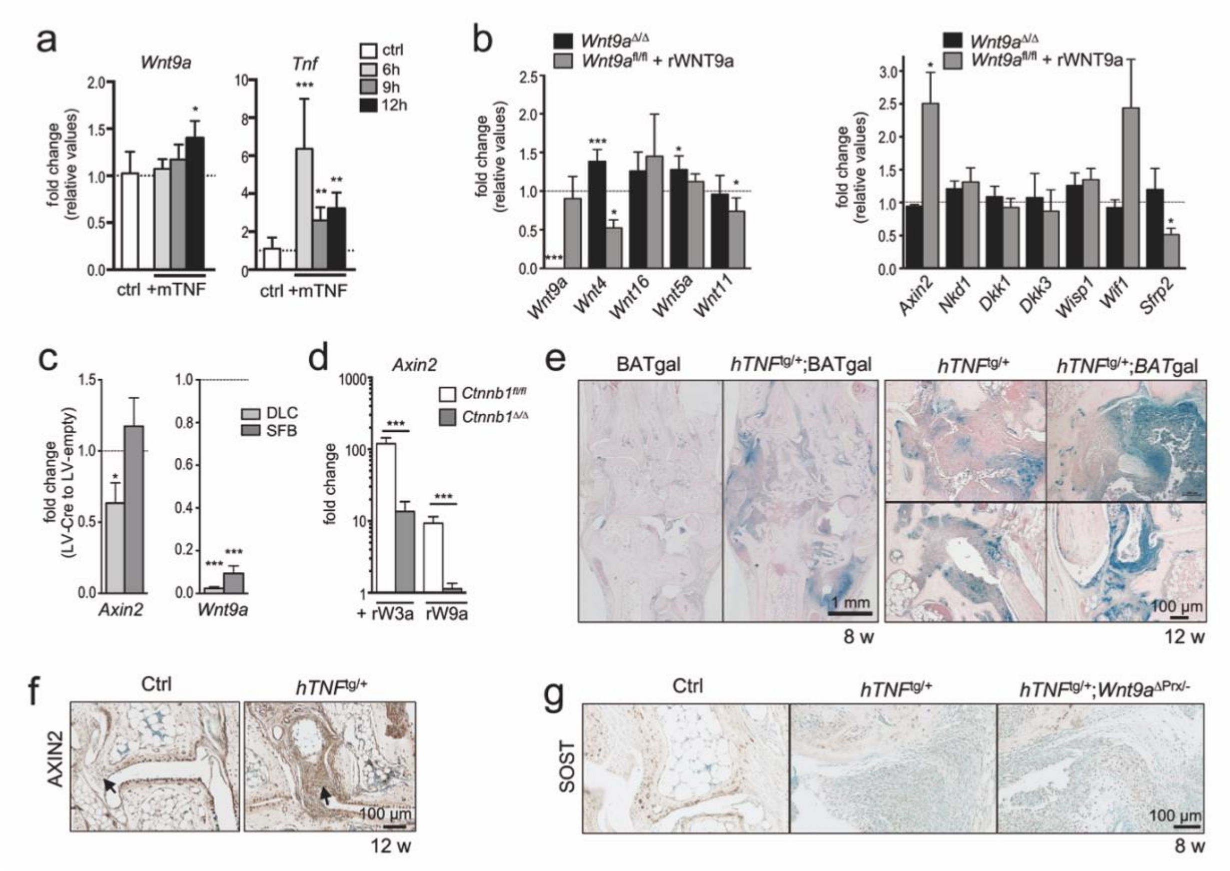

Supplementary Figure S1

Expression analysis of Wnts and Wnt pathway-related genes in cultured SFBs.

a qPCR analysis of short term effects on the expression of Wnt9a and Tnf in response to stimulation with recombinant murine (m) or human (h) TNF for 12 h. **b** qPCR analysis of Wnt genes and Wnt pathway-related genes in *Wnt9a*$^{\Delta/\Delta}$ (*Wnt9a*-deficient) SFBs and *Wnt9a*$^{fl/fl}$ (wild-type) SFBs treated with rWNT9a for five days displayed as fold change relative to untreated control. **c** qPCR analysis of Axin2 and Wnt9a expression in *Wnt9a*-deficient DLCs and SFBs relative to control cells infected with an empty lentivirus. **d** qPCR analysis of Axin2 expression in response to rWNT3a and rWNT9a treatment in *Ctnnb1*$^{fl/fl}$ (wild-type) and β-catenin–deficient (*Ctnnb1*$^{\Delta/\Delta}$) SFBs presented as fold change with a logarithmic scale. **e** β-galactosidase activity staining on sections of hind paws from 8 week-old BATgal and *hTN*F$^{tg/+}$;BATgal and 12 week-old *hTN*F$^{tg/+}$ and *hTN*F$^{tg/+}$;BATgal mice. **f** Immunohistochemical staining for AXIN2 on sections through hind paws of 12 week-old *hTN*F$^{tg/+}$ mice and WT littermate control mice. **g** Immunohistochemical staining for SOST on sections through hind paws of 8 week-old *hTN*F$^{tg/+}$ and *hTN*F$^{tg/+}$;*Wnt9a*$^{\Delta Prx1/-}$ mice. *A-G*: n=3. Two-tailed, unpaired Student's t-test. Data are presented as the mean ± SD. *P*-values: *<0.05, **<0.01, and ***<0.001.

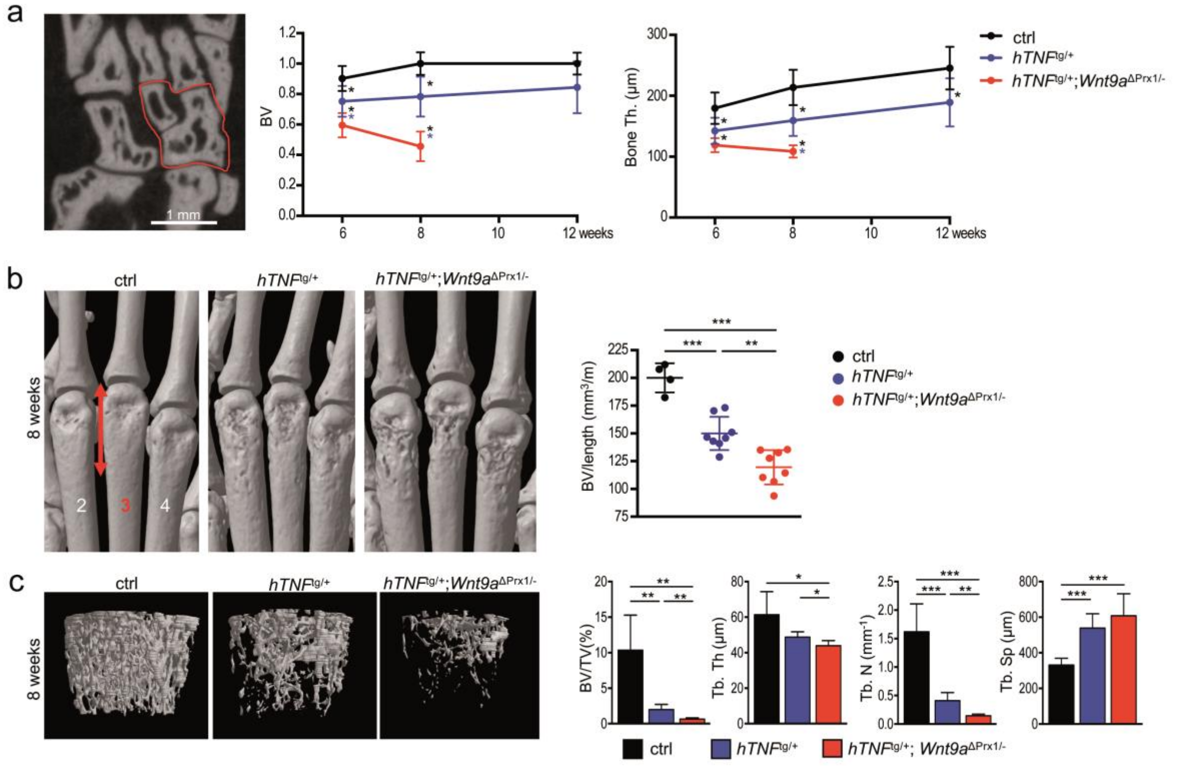

Supplementary Figure S2

Wnt9a-deficiency promotes bone destruction in hTNF transgenic mice.

a Two-dimensional microCT images of tarsal regions with the area (outlined in red), which was used for the quantification of bone volume (BV) and bone thickness (Bone Th.) of ctrl, *hTN*F$^{tg/+}$, and *hTN*F$^{tg/+}$;*Wnt9a*$^{\Delta Prx1/-}$ specimens at the 6-, 8-, and for ctrl and *hTNF*$^{tg/+}$ also at the 12-week time point. Ctrl (6w: n=9; 8w: n=13; 12w: n=9), *hTNF*$^{tg/+}$ (6w: n=9; 8w: n=18; 12w: n=9), and *hTN*F$^{tg/+}$;*Wnt9a*$^{\Delta Prx1/-}$ (6w: n=9; 8w: n=8). Statistical significance of the values against the ctrl are indicated by a black asterisk and against *hTNF*$^{tg/+}$ by a blue asterisk. **b** Representative 3D microCT images of distal metatarsal region of 8-week-old ctrl, *hTN*F$^{tg/+}$, and *hTN*F$^{tg/+}$;*Wnt9a*$^{\Delta Prx1/-}$ specimens. The red double-arrow represents the region of the third metatarsal element used for bone volume quantification displayed in the dot-plot to the right. Ctrl: n=4; *hTN*F$^{tg/+}$: n=8, and *hTN*F$^{tg/+}$;*Wnt9a*$^{\Delta Prx1/-}$: n=8. **c** Representative 3D microCT images of the trabecular bone of the distal femur of 8-week-old male ctrl (n=7), *hTNF*$^{tg/+}$ (n=5), and *hTN*F$^{tg/+}$;*Wnt9a*$^{\Delta Prx1/-}$ (n=4) specimens, and corresponding quantification of bone volume/total volume (BV/TV), trabecular thickness (Tb.Th), trabecular number (Tb.N), and trabecular spacing (Tb.Sp). Data in **a-c** are presented as the mean value ± SD. *P*-values: *<0.05; **<0.01; ***<0.001; ns = not significant.

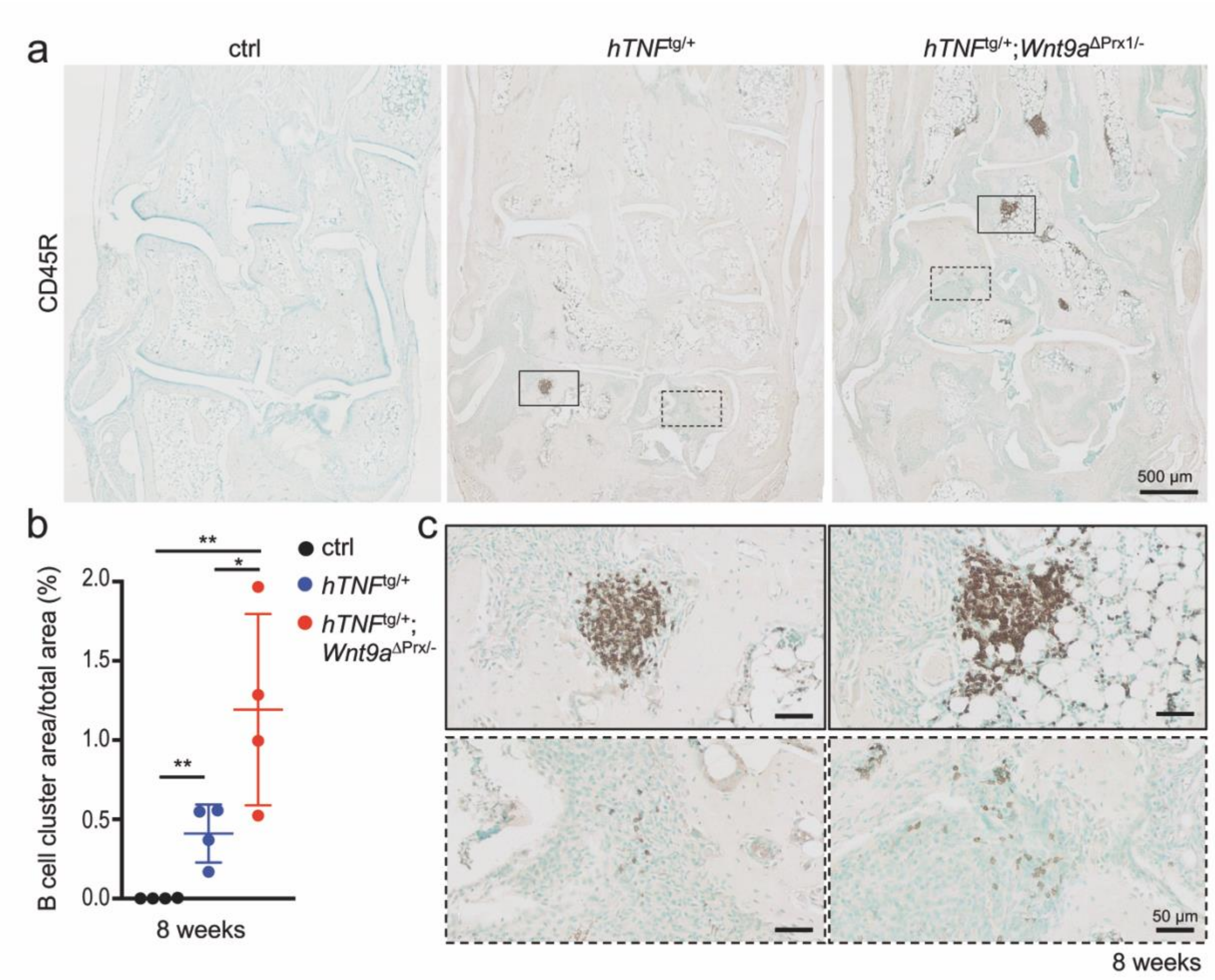

Supplementary Figure S3

Presence of B cell clusters in the hind paws of *hTN*F$^{tg/+}$ and *hTN*F$^{tg/+}$/*Wnt9a*$^{\Delta Prx/-}$ mice.

a Representative images of CD45R stained sections through the hind paw region of 8-week-old ctrl, *hTN*F$^{tg/+}$, and *hTN*F$^{tg/+}$;*Wnt9a*$^{\Delta Prx/-}$ mice. No CD45R positive B cell clusters are present in the control, while these clusters are present in the bone marrow cavity of *hTN*F$^{tg/+}$ and *hTN*F$^{tg/+}$;*Wnt9a*$^{\Delta Prx/-}$ mice (n=4). **b** Quantification of the B cell cluster area to total area showing a statistically significant increase in the *hTN*F$^{tg/+}$;*Wnt9a*$^{\Delta Prx/-}$ mice compared to *hTN*F$^{tg/+}$and control mice (n=4) **P*-value <0.05; ***P*-value <0.01. **c** Magnified images of the regions indicated by the solid and stippled boxed areas in (**a**) showing such a CD45R^{+} B cell cluster in the bone marrow and single CD45R positive cells in a pannus region.

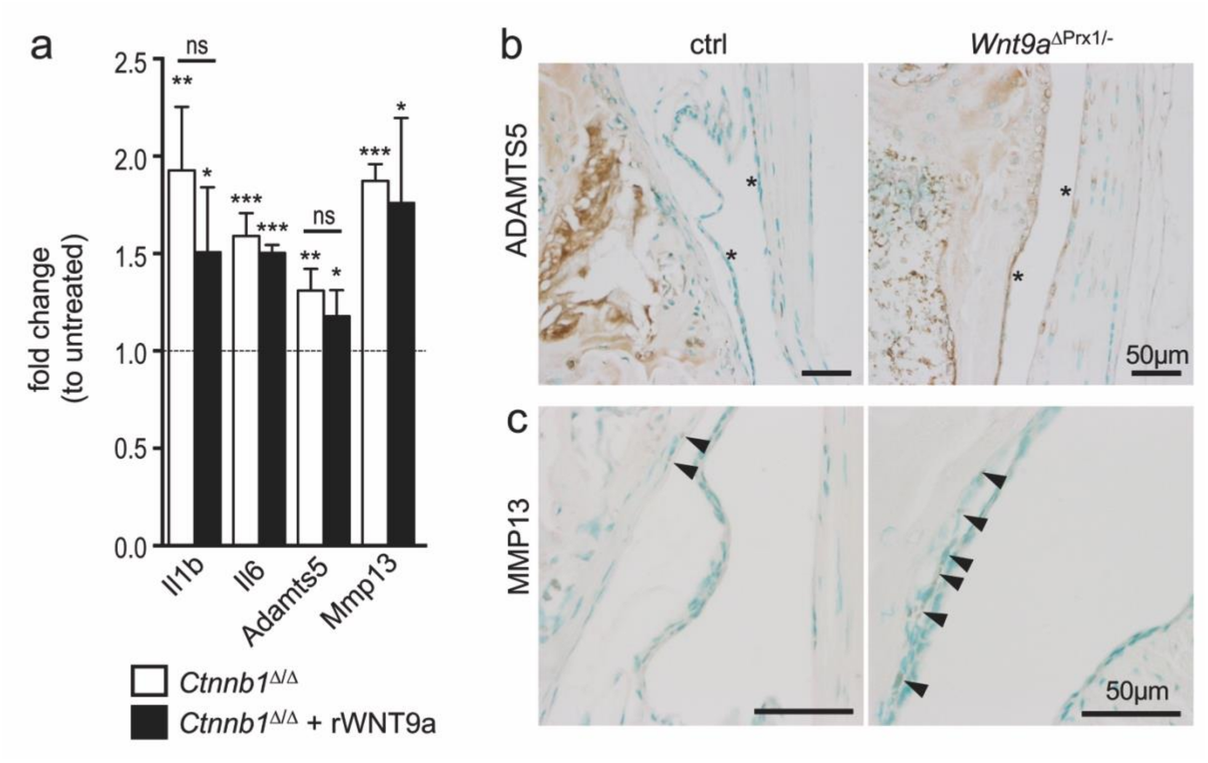

Supplementary Figure S4

Involvement of β-catenin in the negative regulation of Il1b, Il6, Adamts5, and Mmp3 and presence of ADAMTS5 and MMP13 positive synovial cells in *Wnt9a*$^{\Delta Prx/-}$ mice.

a qPCR analysis of Il1b, Il6, Adamts5, and Mmp13 in *Ctnnb1*-deficient SFBs untreated or treated with rWNT9a for five days. Two-tailed, unpaired Student's t-test (n=4). Data are presented as the mean ± SD. *P*-values: *<0.05, **<0.01, and ***<0.001. ns = not significant. **b** Representative images of ADAMTS5 stained sections through the knee region of 6-month old ctrl and *Wnt9a*$^{\Delta Prx/-}$ mice (n=3). The synovial lining layers are marked by asterisks. **c** Representative images of MMP13 stained sections through the knee region of 6-month old ctrl and *Wnt9a*$^{\Delta Prx/-}$ mice. Arrow heads are pointing at MMP13 positive cells within the synovial lining (n=3).

Immunoblots (uncropped):

Corresponding to **Figure 4f**

Loading scheme lanes 1-12, numbers in bracket represent independent biological samples (used for quantification):

1 *Wnt9a*$^{fl/fl}$, 0 min hTNF
2 *Wnt9a*$^{fl/fl}$, 10 min hTNF
3 *Wnt9a*$^{fl/fl}$, 20 min hTNF
4 *Wnt9a*$^{fl/fl}$, 30 min hTNF
5 *Wnt9a*$^{Δ/Δ}$, 0 min hTNF
6 *Wnt9a*$^{Δ/Δ}$, 10 min hTNF
7 *Wnt9a*$^{Δ/Δ}$, 20 min hTNF
8 *Wnt9a*$^{Δ/Δ}$, 30 min hTNF
9 *Wnt9a*$^{fl/fl}$ + rWNT9a, 0 min hTNF
10 *Wnt9a*$^{fl/fl}$ + rWNT9a, 10 min hTNF
11 *Wnt9a*$^{fl/fl}$ + rWNT9a, 20 min hTNF
12 *Wnt9a*$^{fl/fl}$ + rWNT9a, 30 min hTNF

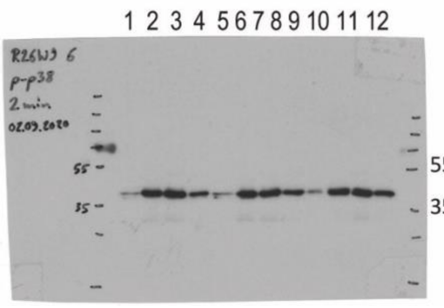

p-P38 (43 kDa)

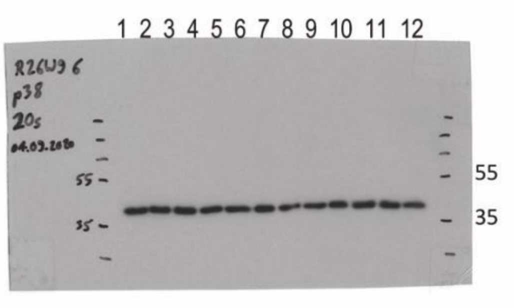

P38 (40 kDa)

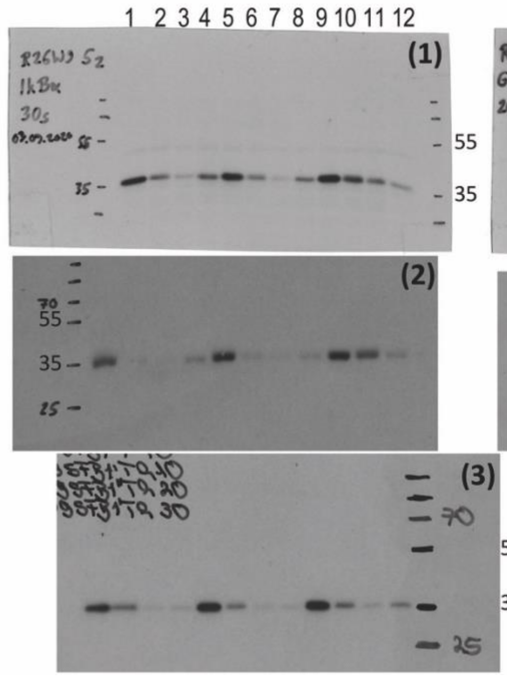

IκBα (39 kDa)

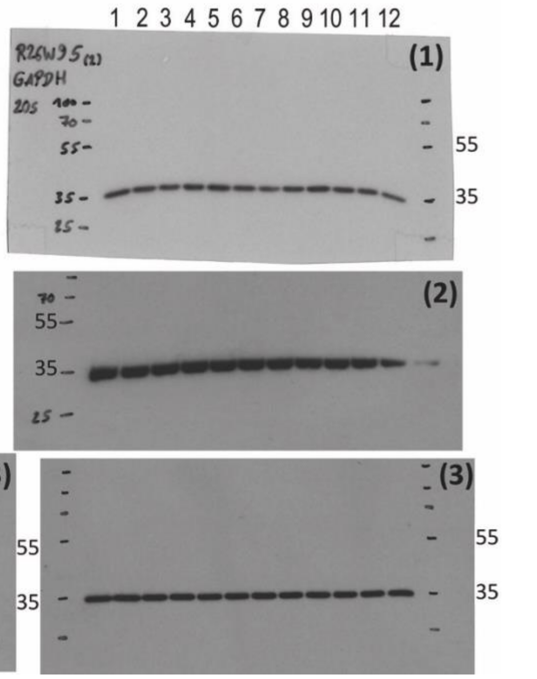

GAPDH (37 kDa)

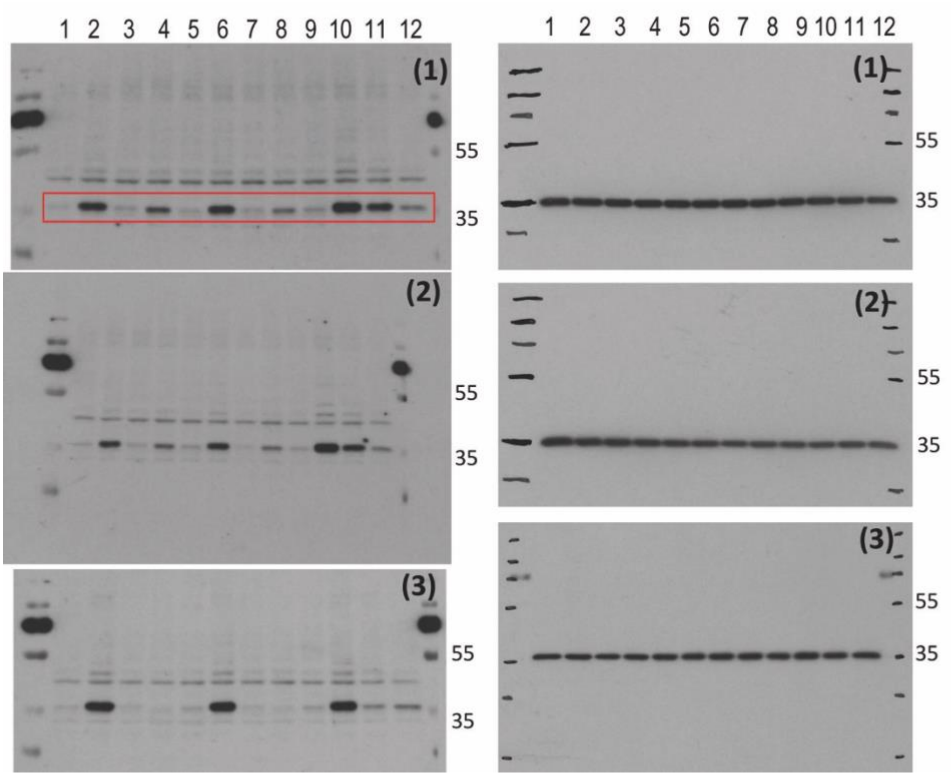

pIκBα (40 kDa)

GAPDH (37 kDa)

Corresponding to **Figure 4g**

Loading scheme lanes 1-4, numbers in bracket represent independent biological samples:

1 SFBs + 0 min rWnt9a
2 SFBs + 10 min rWnt9a
3 SFBs + 20 min rWnt9a
4 SFBs + 30 min rWnt9a

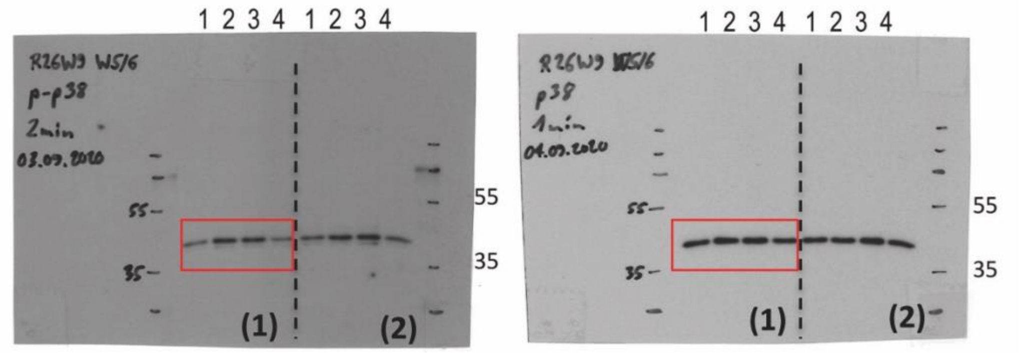

p-P38 (43 kDa)

P38 (40 kDa)

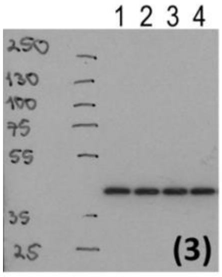

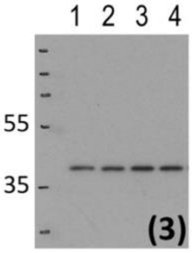

p-P38 (43 kDa) P38 (43 kDa)

Corresponding to **Figure 4h**

Loading scheme lanes 1-4, numbers in bracket represent independent biological samples:

1 SFBs + 0 min rWnt9a
2 SFBs + 10 min rWnt9a
3 SFBs + 20 min rWnt9a
4 SFBs + 20 min rWnt9a

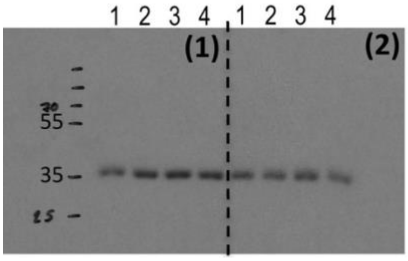

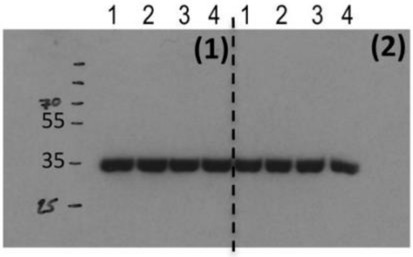

IκBα (39 kDa) GAPDH (37 kDa)

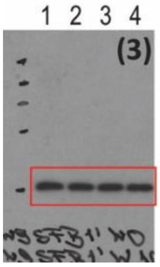

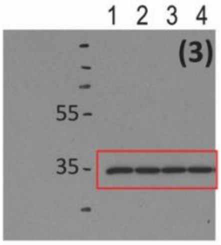

IκBα (39 kDa) GAPDH (37 kDa)

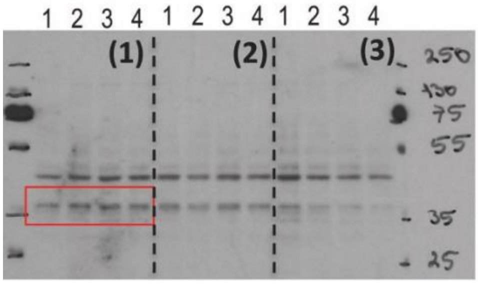

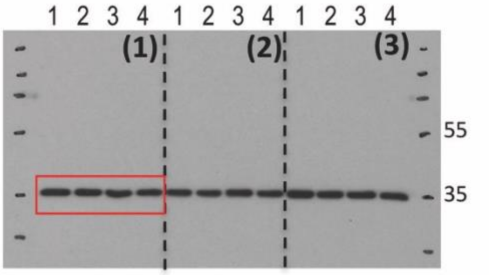

pIκBα (40 kDa) GAPDH (37 kDa)
